# Combination effect of antithrombin and recombinant human soluble thrombomodulin in a lipopolysaccharide induced rat sepsis model

**DOI:** 10.1186/cc8210

**Published:** 2009-12-14

**Authors:** Toshiaki Iba, Etsuro Nakarai, Toshio Takayama, Kenji Nakajima, Tetsumasa Sasaoka, Yoichi Ohno

**Affiliations:** 1 Department of Emergency and Disaster Medicine, Juntendo University, 2-1-1 Hongo Bunkyo-ku, Tokyo, 113-8421, Japan; 2 Pharmaceutical Research Laboratory, Nihon Pharmaceutical Co., LTD., 26 Sumiyoshi-cho Izumisano, Ohsaka, 598-8558, Japan

## Abstract

**Introduction:**

Recombinant human soluble thrombomodulin (rhsTM) is newly developed for the treatment of DIC. The purpose of this study was to evaluate the efficacy of the concomitant administration of rhsTM and antithrombin (AT).

**Methods:**

In the first series, rats were treated with either 62.5, 125, 250 or 500 IU/kg (n = 6, each) of AT or 0.125, 0.25, 0.5 or 1.0 mg/kg (n = 6, each) of rhsTM followed by lipopolysaccharide (LPS) injection. 8 h later, the fibrinogen level was examined. In the second series, TM group was pretreated with 0.25 mg/kg of rhsTM, AT group was pretreated with 125 IU/kg of AT, AT/TM group was pretreated with both AT and rhsTM, and control group was pretreated with saline (n = 7, each). The platelet count, fibrinogen, ALT, LDH and high-mobility group box 1 (HMGB1) levels were measured. In addition, histologic changes in liver were examined. In the third series, survival was calculated up to 24 h.

**Results:**

Both AT and rhsTM produced a linear dose-response with regard to the fibrinogen level, with 125 IU/kg of AT and 0.25 mg/kg of rhsTM producing equivalent effects. The combined administration of AT and rhsTM significantly reduced the decrease in the platelet count and the fibrinogen level (*P *< 0.05, 0.01, respectively). The elevations in ALT and LDH were significantly suppressed in all treatment groups. The HMGB1 level and the histologic changes tended to indicate damage reduction. Survival was significantly better only in AT/TM group (*P *< 0.01).

**Conclusions:**

The coadministration of AT and rhsTM might be effective for the treatment of severe sepsis.

## Introduction

The tight cross-talk between the coagulation system and inflammatory reactions during sepsis has attracted much attention and anticoagulant therapies have been expected to be beneficial not only for septic coagulopathy but also for severe sepsis [1-3]. Two major physiological anticoagulant systems, the activated protein C (APC)-thrombomodulin (TM) system and the antithrombin (AT)-heparan sulphate system, have been extensively studied as research targets.

APC is best known for its natural anticoagulant properties, and a multi-center randomized controlled trial (RCT) previously demonstrated the efficacy of recombinant human APC against severe sepsis [4]; thus, the application of recombinant human APC has been strongly recommended in an international guideline for the treatment of severe sepsis [5]. TM, a cell surface-expressed glycoprotein, is predominantly synthesized by vascular endothelial cells, and is a critical cofactor for the thrombin-mediated activation of protein C (PC). A recombinant human soluble TM (rhsTM) has been recently developed [6], and this new agent has some advantages over APC such as a long *in vivo *half-life and a unique amino-terminal structure exhibiting anti-inflammatory activity including sequestration [7] and cleavage of high-mobility group box 1 (HMGB1) [8], recently identified as a lethal late-phase mediator and is suspected to be closely correlated with the development of disseminated intravascular coagulation (DIC) during sepsis [9]. Furthermore, its efficacy on the treatment of DIC was confirmed in a recent RCT [10].

AT is another natural coagulant that has been extensively studied [11-14]. As the anticoagulant effects of the TM-APC system are expressed through a different mechanism from that of the AT-heparan sulphate system, the additive effects can be reasonably expected. As a single drug is likely to be insufficient to improve the outcome of severe sepsis, the purpose of this study was to examine whether the concomitant administration of AT and rhsTM exerts additive effects resulting in better survival in a sepsis model with DIC.

## Materials and methods

Eight-week-old male Wistar rats (Japan SLC, Shizuoka, Japan) were used in this study. All experimental procedures were conducted after obtaining the approval of the Ethical Committee for Animal Experiments of Juntendo University. All rats were provided with standard rat chow and water *ad libitum*. The rats were anesthetized with sodium pentobarbital (40 mg/kg, intraperitoneally), and systemic inflammation was induced by administering a single injection of lipopolysaccharide (LPS; L4130 0111:B4, Sigma Chemical Co., St. Louis, MO, USA) via the caudal vein at a dose of 20 mg/kg.

### Dose setting study

In the first series, a total of 54 rats were treated with either saline alone (n = 6), human plasma-derived AT (Nihon Pharmaceutical Co., Osaka, Japan) at a dose of 62.5, 125, 250, or 500 IU/kg (n = 6, each dose) or rhsTM (ART-123, Asahi Kasei Pharma Co., Tokyo, Japan) at a dose of 0.125, 0.25, 0.5, or 1.0 mg/kg (n = 6, each dose) followed by LPS injection. Eight hours later, blood samples were obtained from the inferior *vena cava *and the plasma fibrinogen level was measured using clotting methods and the Automated Blood Coagulation Analyzer CS-2000 *i *(Sysmex Co., Kobe, Japan). In addition to the control and treatment groups, the plasma fibrinogen level was also measured in normal rats (n = 6)

### Co-administration study

In the second series, 28 animals were divided into four groups. In the AT group (n = 7), 125 IU/kg of AT was intravenously administered via the caudal vein before LPS injection. In the TM group (n = 7), 0.25 mg/kg of rhsTM was intravenously administered via the caudal vein before LPS injection. In the AT/TM group (n = 7), 125 IU/kg of AT and 0.25 mg/kg of rhsTM were intravenously administered before LPS injection. In the control group (n = 7), animals were given saline alone before the treatment of LPS. At eight hours after the LPS injection, rats were sacrificed under deep anesthesia in an ether chamber. Blood samples were obtained from the inferior vena cava, and the platelet count was determined using a multi-automatic blood cell counter for animals (MICROS abc LC-152, HORIBA, Ltd. Tokyo, Japan). Citrated plasma samples obtained by whole blood centrifugation were stored at -80°C until assay. The plasma fibrinogen level and the levels of the organ damage markers alanine aminotransferase (ALT) and lactate dehydrogenase (LDH) were measured in the samples. The enzymatic activity of LDH was measured using an LDH-J kit (Wako Chemicals, Osaka, Japan). In the same samples, the HMGB1 level was measured using an ELISA kit (Shino-test, Tokyo, Japan).

In the same series, a portion of the liver was excised and the specimens were fixed in 10% buffered formalin, embedded in paraffin, stained with H&E, and examined using light microscopy. In addition to the control and treatment groups, the same measurement was performed in normal rats (n = 7).

### Survival

In the third series, survival was calculated up to 24 hours after LPS injection in the control (n = 23), AT (n = 22), TM (n = 22) and AT/TM groups (n = 22).

### Statistics

All data were expressed as the mean ± standard deviation. A statistical analysis was carried out using the SAS program (version 8.02, SAS Institute, Cary, North Carolina, USA). Comparison between the normal and control groups were examined using the Welch's t-test for non-parametric analysis. Comparisons between the treatment groups and the control group were carried out using the Dunnett's or Steel test. Survival was examined using a log-rank test. Statistical differences were deemed significant at a level of *P *< 0.05.

## Results

**Figure 1:**
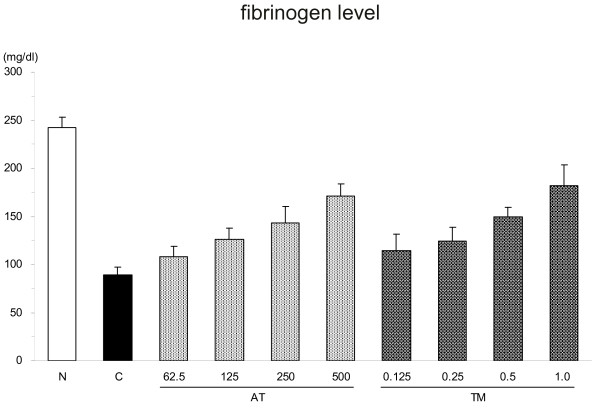
Comparison of fibrinogen levels. The plasma fibrinogen level decreased significantly decreased at eight hours after lipopolysaccharide (LPS) injection, and this decrease was suppressed by treatment with either antithrombin (AT) or thrombomodulin (TM) in a dose-dependent manner (n = 6, for each dose). The fibrinogen levels were 126.6 ± 11.1 mg/dL and 124.2 ± 14.7 mg/dL after pre-treatment with 125 IU/kg of AT and 0.25 mg/kg of TM, respectively. N = normal; C = control.

The dose setting study revealed the dose-response effects for the maintenance of fibrinogen levels after LPS injection in both the AT and rhsTM treatment groups. The plasma fibrinogen level was 126.6 ± 11.1 mg/dL after treatment with125 IU/kg of AT, or approximately half of the normal level; treatment with 0.25 mg/kg of rhsTM produced a comparable effect (Figure 1). Thus, 125 IU/kg of AT and 0.25 mg/kg of rhsTM was utilized in subsequent studies.

**Figure 2:**
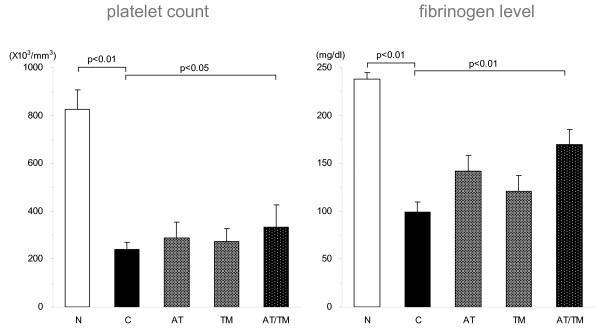
Changes in coagulation markers. The platelet count had decreased significantly at eight hour after lipopolysaccharide (LPS) injection in the control group (n = 7). This depletion was slightly suppressed in both the antithrombin (AT) and thrombomodulin (TM) groups (n = 7 each); however, the differences were not statistically significant. Meanwhile, the platelet count in the AT/TM group was significantly higher in the AT/TM group (n = 7). Similarly, the fibrinogen level was significantly depressed at eight hours after LPS injection in the control group (n = 7) and this depletion was suppressed in the AT and TM groups; however, the differences were not statistically significant. The level was best maintained in the AT/TM group (*P *< 0.01, n = 7). N = normal; C = control.

In the co-administration study, the platelet counts and fibrinogen levels decreased significantly after LPS injection. These reductions were suppressed in both the AT and rhsTM groups; however; the differences were not statistically significant in these groups. In contrast, the AT/TM group showed a significant reduction in these haemostatic markers (*P *< 0.05 for the platelet count, *P *< 0.01 for the fibrinogen level; Figure 2).

**Figure 3:**
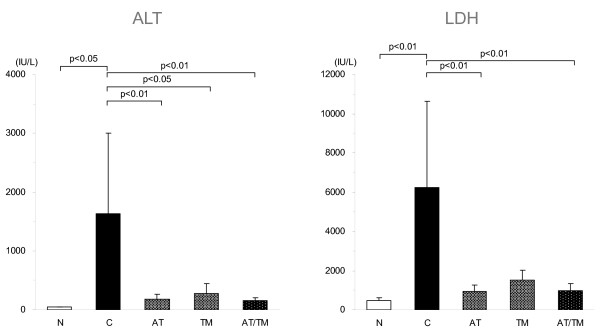
Changes in organ dysfunction markers. The elevated level of alanine aminotransferase (ALT) at eight hours after lipopolysaccharide (LPS) injection in the control group (n = 7) was significantly suppressed in the antithrombin (AT, n = 7, *P *< 0.01), recombinant human thrombomodulin (TM, n = 7, *P *< 0.05), and AT/TM groups (n = 7, *P *< 0.01). The elevated lactate dehydrogenase (LDH) level in the control group at eight hours was significantly suppressed in the AT and AT/TM groups (*P *< 0.01, each). N = normal; C = control.

The levels of ALT and LDH were significantly elevated after LPS injection, and the elevation of ALT was significantly suppressed in all the treatment groups (*P *< 0.05 in TM, *P *< 0.01 in AT and AT/TM; Figure 3, left). Similarly, the elevation of LDH was significantly suppressed in the AT and AT/TM groups (*P *< 0.01 in each group; Figure 3, right). However, a significant difference was not recognized between the AT and AT/TM groups.

**Figure 4:**
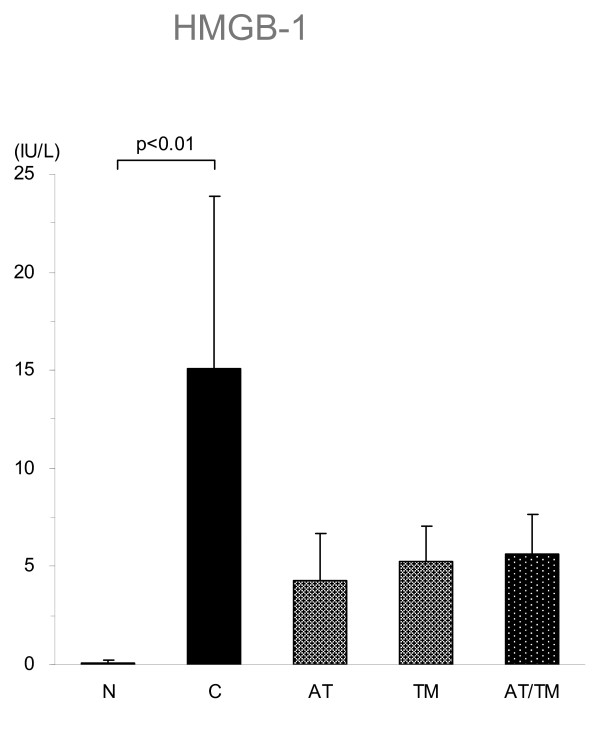
Changes in HMGB1. The high-mobility group box 1 (HMGB1) level increased at 8 h after the treatment of lipopolysaccharide (LPS) treatment in the control group (n = 7). The levels were lower in antithrombin (AT, n = 7), recombinant human soluble thrombomodulin (TM, n = 7) and AT/TM groups (n = 7) and were approximately one-third of that in the control group; however, the differences were not rstatistically significant (*P *< 0.1, each). N = normal; C = control.

The HMGB1 level exceeded 15.0 ng/mL at eight hours after LPS injection. This elevation was suppressed in all the treatment groups to a level approximately one-third of that in the control group; however, the differences between each group and the control group were not statistically significant (Figure 4).

**Figure 5:**
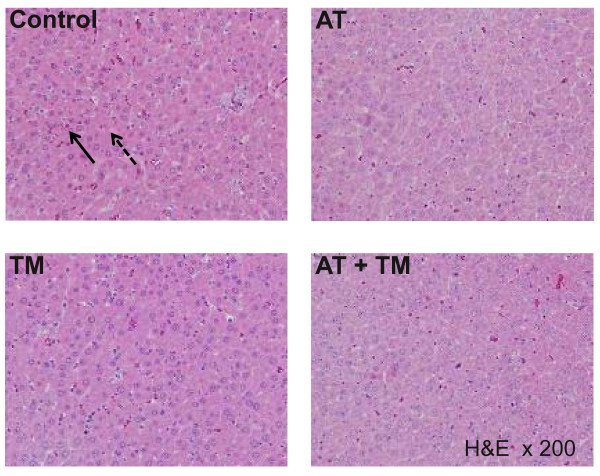
Histological analysis of the liver. Representative photographs (200× magnification; hematoxylin and eosin (H&E) stain) of liver sections taken eight hours after the injection of lipopolysaccharide (LPS) in partially hepatectomized rats that had been treated with saline (control), antithrombin (AT), recombinant human soluble thrombomodulin (TM), or AT and TM (AT/TM). The solid arrow points to leukocyte infiltration, and the hatched arrow points to necrotic hepatocytes.

The histological changes after LPS injection were characterized by inflammatory cell infiltration, hemorrhagic changes and focal necrosis in the midzone and periportal regions of the liver at eight hours after LPS injection. These histological alterations were attenuated by pre-treatment with either AT, rhsTM or a combination of AT and rhsTM. Although the histological changes were not prominent, the changes were further confirmed by a serological analysis (Figure 5).

**Figure 6:**
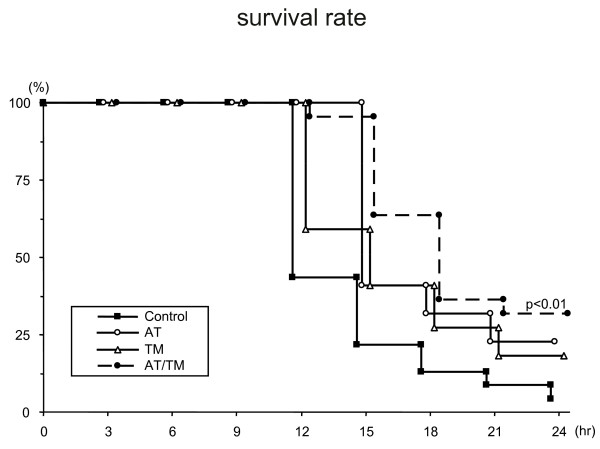
Survival after LPS injection. The Kaplan-Meier survival curve shows survival for a maximum of 24 hours after lipopolysaccharide (LPS) injection in the control (n = 23), antithrombin (AT; n = 22), recombinant human soluble thrombomodulin (TM; n = 22) and AT and TM (AT/TM; n = 22) groups. Seven of 22 rats (31.8%) survived in the AT/TM group, while only 1 of 23 rats (4.3%) survived in the control group at 24 hours after LPS injection; this difference between the groups was significant.

A Kaplan-Meier survival curve was calculated using data obtained for a maximum of 24 hours after LPS injection; survival started to decrease beginning at 12 hours after LPS injection, and at 24 hours only 1 of 23 rats (4.4%) was alive in the control group, whereas 7 of the 22 rats (31.8%) were still alive in the AT/TM group; the difference between the groups was significant (*P *< 0.01; Figure 6).

## Discussion

Accumulating evidence suggest that many anticoagulants have effects on inflammation [15]. For example, we previously reported the efficacy of danaparoid sodium [16], recombinant APC [17] and AT [12] in a similar rat sepsis model. However, none of these drugs achieved a satisfactory result and the combined administration of antithrombotic agents may provide a multipronged approach to the treatment of severe sepsis. Consequently, we planned the present experiment to examine the effects of a combination of AT and rhsTM.

With regard to the dose settings for both agents, because the primary purpose of this study was not to compare AT and rhsTM but to examine the additive effects of these agents, the dose of AT was set at a relatively low value. Actually, we previously reported that a dose of 125 IU/kg of AT enabled recovery to a value with the normal range and revealed the additive effects of AT and danaparoid sodium [18].

The effects of rhsTM on DIC were previously examined in a multi-center RCT, and the resolution of DIC was significantly better in the group treated with rhsTM than in the group treated with unfractionated heparin [11]. As the patients with resolved DIC had a lower mortality rate in this RCT, the correction of the coagulation abnormalities was suggested to lead to improved survival. In addition, other experiments reported that the effects of rhsTM were not only limited to DIC but that beneficial effects were also obtained against severe sepsis and other inflammatory responses [19,20]. Therefore, a clinical trial to determine the effects of rhsTM on severe sepsis is now underway.

AT was expected to have favorable effects against severe sepsis, but it was denied in a large-scale multi-center RCT [11]. However, as a subgroup analysis reported beneficial effects in septic DIC patients [13], Hoffmann and colleagues [14] performed a small-sized RCT with an enrollment of 40 patients. This study demonstrated a better maintenance of haemostatic markers such as prothrombin time and fibrinogen level.

The mortality rate for severe sepsis is still high and a single drug may be inadequate to obtain a favorable outcome [21]; therefore, we planned to examine the effects of the concomitant use of AT and rhsTM. Theoretically, the combination of AT and rhsTM could possibly exert additive or synergistic effects because the anticoagulant mechanisms differ for these agents. As a result, the changes of platelet count and the fibrinogen levels were suppressed significantly only in the AT/TM group. However, as for the improvement in the organ dysfunction markers, there was no statistically significant difference in the ALT or LDH levels between AT and AT/TM group in this study. As for why we could not see the additive effects, we speculate that because AT alone had already suppressed the elevation of LDH significantly, and the effects of rhsTM might be concealed.

With respect to the survival, it was better maintained in the AT/TM group compared with that in the control group. However, the significant difference was not seen between the AT group and AT/TM group, or rhsTM group and AT/TM group.

The mechanism of action responsible for these effects was not fully elucidated; however, other than the maintenance of coagulation disorders, the exertion of anti-inflammatory effects may contribute to the end result. To address this issue, the changes in the HMGB1 level were examined. HMGB1 is an intranuclear protein that was originally identified as a DNA-binding protein, [22], but has been recognized as a late-phase mediator during sepsis [23]. HMGB1 is also known to act as a pro-coagulant [24] as well as a pro-inflammatory mediator for septic organ dysfunction [25,26]. However, whether anticoagulant therapy can regulate this so-called lethal mediator remains uncertain [27,28]. Although the HMGB1 level tended to decrease with AT and rhsTM pre-treatment in this study, the role of this mechanism in organ protection and the improvement in survival is still uncertain because the decrease was not significant.

Other than the above mechanisms, as the pathologic findings suggested a lower level of inflammatory cell infiltration and necrotic changes after treatment, these mechanisms might contribute to the maintenance of organ protection. However, the changes were not remarkable probably because of the early timing of the sampling, so further study is needed to clarify the mechanism of action.

## Conclusions

Although the additive effects of AT and rhsTM were recognized with regard to the changes in coagulation markers, the organ damage levels were similar among the AT, TM, and AT/TM groups. Survival was significantly better in the AT/TM group.

## Key messages

• The changes in coagulation abnormalities were reduced by the coadministration of AT and rhsTM.

• The additive effects of AT and rhsTM were recognized in the survival of septic rats.

## Abbreviations

ALT: alanine aminotransferase; APC: activated protein C; AT: antithrombin; DIC: disseminated intravascular coagulation; ELISA: enzyme-linked immunosorbent assay; H&E: hematoxylin and eosin; HMGB1: high-mobility group box 1; LDH: lactate dehydrogenase; LPS: lipopolysaccharide; RCT: randomized controlled trial; rhsTM: recombinant human thrombomodulin.

## Competing interests

The authors state that TI, EN and TTT have no conflict of interest. KN, TS and YO are the researchers of the Research Laboratory of Nihon Pharmaceutical.

## Authors' contributions

TI designed the study and wrote the manuscript. EN and TT processed the data. KN, TS and YO performed the experiment and collected the data. All authors read and approved the final manuscript.
